# Application Value of Radiomic Nomogram in the Differential Diagnosis of Prostate Cancer and Hyperplasia

**DOI:** 10.3389/fonc.2022.859625

**Published:** 2022-04-14

**Authors:** Shaogao Gui, Min Lan, Chaoxiong Wang, Si Nie, Bing Fan

**Affiliations:** ^1^Department of Radiology, Jiangxi Provincial People’s Hospital, The First Affiliated Hospital of Nanchang Medical College, Nanchang, China; ^2^Department of Orthopedics, Jiangxi Provincial People’s Hospital, The First Affiliated Hospital of Nanchang Medical College, Nanchang, China

**Keywords:** prostate cancer, hyperplasia, magnetic resonance imaging, radiomic, textural features

## Abstract

**Objective:**

Prostate cancer and hyperplasia require different treatment strategies and have completely different outcomes; thus, preoperative identification of prostate cancer and hyperplasia is very important. The purpose of this study was to evaluate the application value of magnetic resonance imaging (MRI)-derived radiomic nomogram based on T2-weighted images (T2WI) in differentiating prostate cancer and hyperplasia.

**Materials and Methods:**

One hundred forty-six patients (66 cases of prostate cancer and 80 cases of prostate hyperplasia) who were confirmed by surgical pathology between September 2019 and September 2019 were selected. We manually delineated T2WI of all patients using ITK-SNAP software and radiomic analysis using Analysis Kit (AK) software. A total of 396 tumor texture features were extracted. Subsequently, the effective features were selected using the LASSO algorithm, and the radiomic feature model was constructed. Next, combined with independent clinical risk factors, a multivariate Logistic regression model was used to establish a radiomic nomogram. The receiver operator characteristic (ROC) curve was used to evaluate the prediction performance of the radiomic nomogram. Finally, the clinical application value of the nomogram was evaluated by decision curve analysis.

**Results:**

The PSA and the selected imaging features were significantly correlated with the differential diagnosis of prostate cancer and hyperplasia. The radiomic model had good discrimination efficiency for prostate cancer and hyperplasia. The training set (AUC = 0.85; 95% CI: 0.77–0.92) and testing set (AUC = 0.84; 95% CI: 0.72–0.96) were effective. The radiomic nomogram, combined with the radiomic characteristics of MRI and independent clinical risk factors, showed better differentiation efficiency in the training set (AUC = 0.91; 95% CI: 0.85–0.97) and testing set (AUC = 0.90; 95% CI: 0.81–0.99). The decision curve showed the clinical application value of the radiomic nomogram.

**Conclusion:**

The radiomic nomogram of T2-MRI combined with clinical risk factors can easily identify prostate cancer and hyperplasia. It also provides suggestions for further clinical events.

## Introduction

Prostate cancer (PCa) is one of the most common malignant tumors in men, and it is also the second most common cause of death related to cancer (1). The overdiagnosis and overtreatment of prostate cancer is a major concern in modern prostate cancer management (2). Noninvasive diagnostic methods commonly used in clinics include prostate-specific antigen (PSA) detection, digital rectal examination, and magnetic resonance imaging (MRI). However, PSA is not accurate in predicting prostate cancer, and it is also increased in benign prostatic hyperplasia (BPH) and granulomatous inflammation of prostatitis (3). Recently, some scholars have found that PSA density is also useful in identifying patients with elevated PSA due to PCa rather than intraprostatic inflammation (4). Digital rectal examination can only initially determine the size, surface, and texture of the prostate but cannot make an accurate diagnosis. Prostate puncture biopsy is the most important method for the pathological diagnosis of prostate cancer. In most guidelines, such as AUA, EAU, and NCCN, the indication of prostate puncture is PSA >3 ng/ml (5). Transrectal ultrasound (TRUS)-guided biopsy is a common diagnostic method if a patient has an elevated PSA. However, in clinical application, we found that some patients had a series of side effects due to TRUS-guided biopsy, including pain and hematuria, and some patients need to be hospitalized for observation. This is a warning that TRUS-guided biopsy should not be used casually to alleviate the suffering of patients. Compared with traditional PSA detection and TRUS-guided biopsy, MRI has the advantage of high soft-tissue resolution, which can clearly distinguish the anatomical structure of the prostate and provide information about the location, size, and surrounding invasion of PCa lesions (6). Therefore, it is widely used in PCa diagnosis (7, 8). Magnetic resonance spectrum (MRS) has a certain accuracy in the diagnosis of PCa, prostatitis, and BPH, and metabolic pattern can be used as an auxiliary means of conventional imaging in the diagnosis of PCa, prostatitis, and BPH. Magnetic resonance dynamic contrast enhancement (DCE-MRI) quantitative parameter analysis has a high value in differentiating PCa from BPH. When MRI diagnosis is difficult, clinical use of the Prostate Imaging Reporting and Data System (PI-RADS) is increasing. However, its diagnostic effectiveness varies based on each radiologist, and the consistency of each report mainly depends on the radiologist’s level of experience and learning (9). In recent years, transperineal ultrasound magnetic resonance cognitive fusion prostate-targeted puncture has been gradually used, which has a higher diagnostic rate than a systematic puncture.

Radiomics can deeply mine and analyze image information, extract many phenotypic features, and deeply screen and classify these phenotypic features through machine learning, which can objectively and quantitatively reflect the heterogeneity of tumors. Many advanced image processing methods are used to quantify the data, including high-order texture analysis and morphological feature analysis (10). At present, radiomics has been applied to the analysis of multiple organs in humans and has good clinical value, such as the diagnosis, stage, efficacy, and prognosis evaluation of lung cancer, breast cancer, and brain astrocytoma (11, 12). Nomograms have been widely used in the diagnosis, treatment, and survival evaluation of cancer patients (13). This recent review mentioned that when AI is applied to diagnostic imaging, it shows excellent accuracy in detecting prostate lesions and predicting patient survival and treatment response (14). The purpose of this study was to establish and validate a combined clinical-imaging model and radiomic nomogram based on T2-MRI imaging features and clinical risk factors for noninvasive differentiation of PCa and BPH.

## Materials and Methods

### Clinical Data

A total of 146 patients with prostate nodules who underwent a 3.0-T MR examination in Jiangxi Provincial People’s Hospital from September 2019 to September 2020 were retrospectively analyzed. The study was approved by the hospital ethics committee, and all patients signed informed consent. All patients underwent TRUS-guided biopsy or radical prostatectomy, and pathological and clinical data were obtained. The clinical manifestations include dysuria, frequency of urination, urgency of urination, or elevated PSA (>3 ng/ml). All patients were given PI-RADS scores according to the recommendation of the PIADS. Inclusion criteria are as follows: (1) pathologically confirmed patients after TRUS-guided biopsy or radical prostatectomy; (2) preoperative MR examination was performed with the same equipment and sequence. Exclusion criteria are as follows: (1) Before the MR examination, patients had undergone biopsy, surgery, radiotherapy, or endocrine therapy; (2) The nodule volume is too small (maximum diameter <3 mm), and the lesion boundary is difficult to delineate; and (3) There were artifacts in MR images, which affected the segmentation of lesions. Among the 170 patients, 19 had motion artifacts and 6 had lesions too small to accurately delineate the region of interest (ROI). Ultimately, 146 patients were included in the study (66 prostate cancer and 80 prostate hyperplasia).

### Examination Methods

A Siemens 3 T (MAGNETOM Skyra, Siemens Healthcare, Erlangen, Germany) MR scanner with 16-channel pelvic phased-array coils was used. The center of the coil aligns with the symphysis pubis. Fast spin-echo (FSE) sequence was used in horizontal T2WI, TR4570 ms, TE89 ms, FOV20 mm × 20 mm, matrix 276 × 238, slice thickness 3.0 mm, layer spacing 0 mm, 3 times excitation. Other scan sequences include sagittal T2WI, coronal T2WI, transverse T1WI, and DWI (*b* = 0, 1,000, 2,000 s/mm^2^).

### Feature Extraction

The images were imported into the image software (ITK-SNAP, http://www.itksnap.org/pmwiki/pmwiki.php) in DICOM format. Two radiologists with 5 years of experience in prostate cancer diagnosis (Doctors A and B) manually segmented areas of interest on T2WI and blinded them to pathological results. First, the two doctors analyzed 20 random images to assess repeatability between groups. Doctor A then repeated the same procedure. ICC greater than 0.8 indicated good consistency of feature extraction, and the rest of the image segmentation was performed by Doctor A. When drawing the T2 image of each tumor, we should select the largest section of the lesion and delineate the ROI along the lesion boundary. During the operation, the image should be enlarged, and the edges of the lesion should be avoided. The ROI should be manually delineated in all layers of the lesion and finally merged into a three-dimensional ROI. ROI should be drawn as close to the edge of the tumor as possible and exclude edema, necrosis, and calcification. The original image and the segmented ROI file should be imported into the AK software (Artificial Intelligence Kit V3.0.0.R, GE Healthcare) at the same time. A total of 396 image quantitative feature parameters were extracted, including histogram, morphology, Haralick feature, gray-level co-occurrence matrix (GLCM), run-length matrix (RLM), and gray-scale region matrix. Z-score data normalization is applied to the values of each feature to eliminate the different characteristics in the extraction value scale.

### Feature Selection and Signature Construction

Feature selection and model construction were carried out in the training group. First, Spearman’s method was used to calculate the redundancy between the feature parameters, and the features whose correlation was greater than 0.9 were retained. Second, we employed the maximum-relevance minimum-redundancy (m-RMR) algorithm to select the features by maximizing the correlation between selected features and differentiating benign and malignant, eliminating the redundancy between features. Next, the least absolute shrinkage and selection operator (LASSO) method was employed to further select the most useful features using a penalty parameter, tuning *λ*. We chose the optimal *λ* based on the minimum criteria according to tenfold crossvalidation. The radiomic signature (Radscore) was then calculated for each case *via* a linear combination of selected features that were weighted by respective coefficients.

### The Radiomic Nomogram Construction

Multivariate logistic regression analysis was used to screen for independent predictors of PCa and BPH, including potential predictors such as imaging features and clinical risk factors. To provide a quantitative tool for clinicians to individually distinguish PCa and BPH, we constructed a radiomic nomogram with both radiomic and clinical features based on a multiple logistic regression model. The discriminant performance of the radiomic nomogram was quantified, and the Radscore for each patient in the testing data was calculated using formulas derived from the training data. Calibration and Hosmer–Lemeshow tests were performed in addition to AUC calculations. Decision curve analysis (DCA) was performed by quantifying the net benefits of the training set and test set of the combined model under different threshold probabilities.

### Data Analysis

All statistical analyses were performed using R (Version:3.4.4) software. LASSO regression is implemented using the “GLmnet” package. In a 7:3 ratio, 103 lesions were selected as the training group and 43 lesions as the test group. *t*-Test for continuous variables and Fisher’s exact test for categorical variables were used to detect differences in clinical features between the PCa and BPH groups. After statistical treatment, the difference was statistically significant (*p* < 0.05). We used some specific indicators, such as accuracy sensitivity and specificity, to evaluate the predictive effect of the model.

## Results

### Patient Characteristics

There were 47 prostate cancer and 56 prostatic hyperplasia patients in the training group and 19 prostate cancer and 24 prostatic hyperplasia patients in the test group. In terms of clinical factors, the univariate logistic analysis showed that PSA was a significant factor in predicting PCa. Multivariate Logistic regression analysis showed that PSA and the radiomic signature were significantly different (all *p* < 0. 05), the age turned to be insignificant, as shown in **Table 1**.

**Table 1:** Demographic characteristics in the training and validation sets.

	Training set (*n* = 103)		*p*-value	Testing set (*n* = 43)		*p*-value
	Ca	BHP		Ca	BHP	
Number	47	56		19	24	
Age	71 ± 7.2	53.4 ± 11.8	0.7859	67.4 ± 10.7	69.9 ± 10.1	0.4301
PSA	65.9 ± 70.7	16.5 ± 27.8	<0.0001	62.1 ± 64.2	11 ± 16.6	0.0001
Radscore median [IQR]	−0.7 [−1.8, −0.2]	0.7 [−0.1, 1.3]	<0.0001	1.2 [0, 2]	−0.5 [−1.2, 0.0]	0.0001

### Performance Outcomes for the Clinical Prediction Model

A Logistic regression classifier was established according to the selected clinical features, and a clinical model was established to identify PCa and BPH. The differential effectiveness of this clinical model mainly includes the following performance metrics. For the training set, the AUC was 0.80 (95% CI, 0.72–0.89), while the sensitivity, specificity, and accuracy rates were 72.3%, 80.3%, and 76.7%, respectively. For the testing data, the AUC was 0.74 (95% CI 0.56–0.91), while the sensitivity, specificity, and accuracy rates were 47.3%, 91.6%, and 72.1%, respectively (**Table 2**).

**Table 2:** Predictive performance outcomes of the radiomic nomogram, radiomic algorithm, and clinical model.

Group	Model	Accuracy	95% CI	Sensitivity	Specificity
Training	Clinical	0.767	[0.72; 0.89]	0.723	0.803
	Radiomics	0.796	[0.77; 0.92]	0.702	0.875
	Nomogram	0.883	[0.85; 0.97]	0.765	0.982
Validation	Clinical	0.721	[0.56; 0.91]	0.473	0.916
	Radiomics	0.744	[0.72; 0.96]	0.736	0.750
	Nomogram	0.860	[0.81; 0.99]	0.933	0.821

### Construction and Assessment of the Radiomic Signature

**Figure 1:**
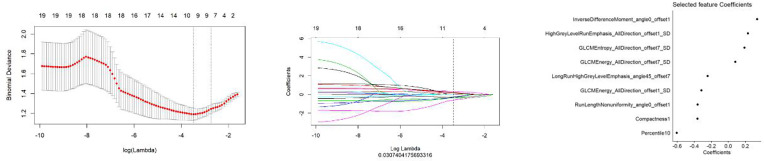
The establishment of LASSO regression model. (Left) Curve of binomial deviation of MR-derived radiomic model varying with parameter *λ*. The vertical axis is binomial deviation. The horizontal axis represents the log (*λ*) value. The number above represents the number of selected features, and the *λ* at the minimum binomial deviation of the model is the optimal value (the curve of the image group characteristic coefficient of the vertical dotted line). (Middle) MR model changing with *λ*. The number above indicates the number of features filtered out. (Right) Imaging features screened by MR model.

**Figure 2:**
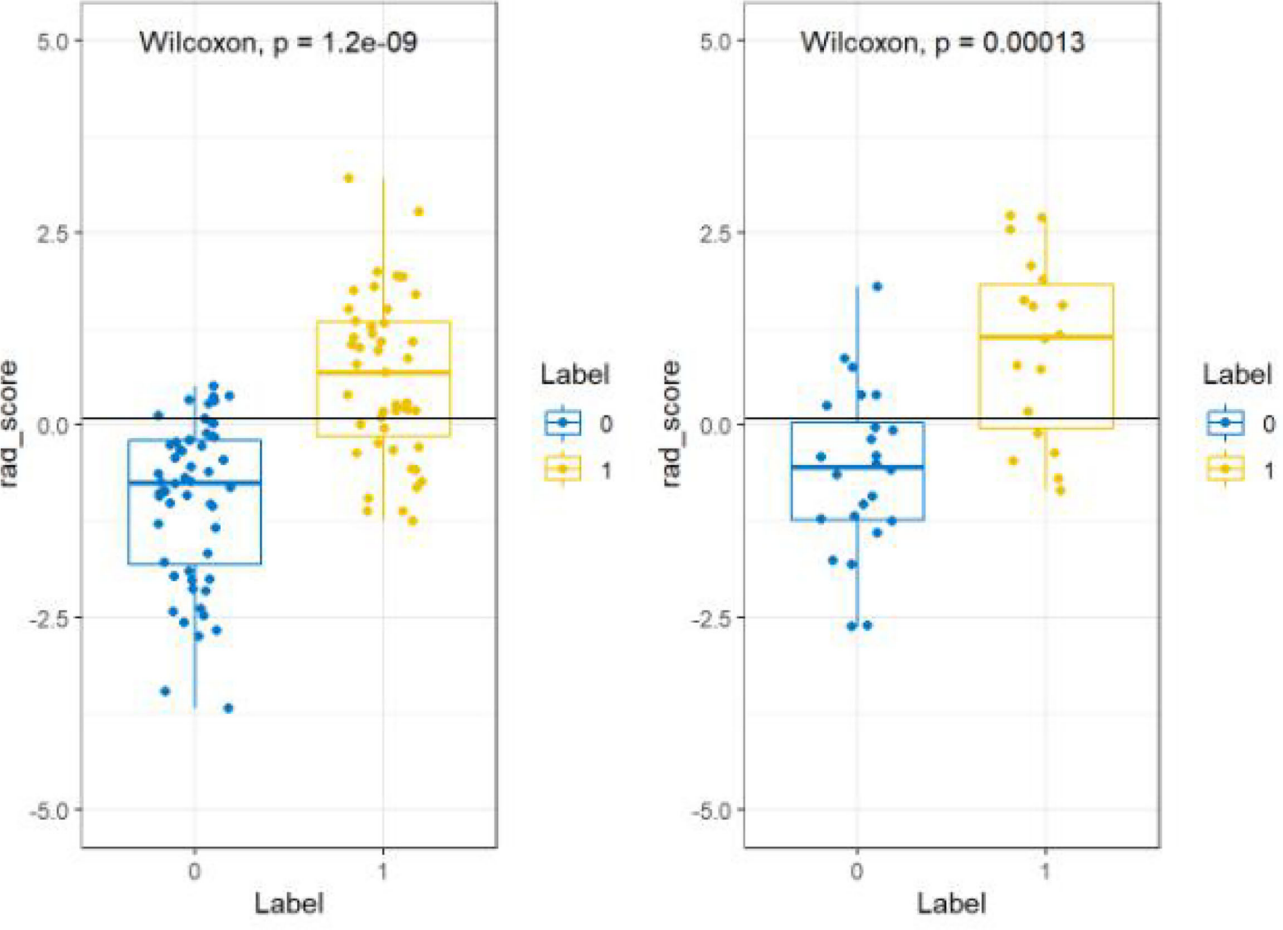
Radiomic labels used in the group model. Comparison of imaging score between MR model training set (left) and rest set (right). The blue label is prostatic hyperplasia, and the yellow label is prostate cancer.

All features were reduced to 9 potential predictors based on 103 patients in the training set using the LASSO algorithm and 10-fold crossvalidation to build a radiomic signature model (**Figure 1**). Our results show that the radiomic group features have good predictive performance for both the training and testing sets. In the two groups, AUC values were 0.91 and 0.90, accuracy was 79.6% and 74.4%, specificity was 87.5% and 75.0%, and sensitivity was 70.2% and 73.6%, respectively (**Table 2**). These selected features were used to calculate the Radscore. The formula is the same as the Chu (15) method. There were significant statistical differences in the Radscore for both the training and testing sets. This suggests that radiomic signatures are closely related to the differential diagnosis of PCa and BPH, as shown in **Figure 2**.

### Construction and Assessment of the Radiomic Nomogram

**Figure 3:**
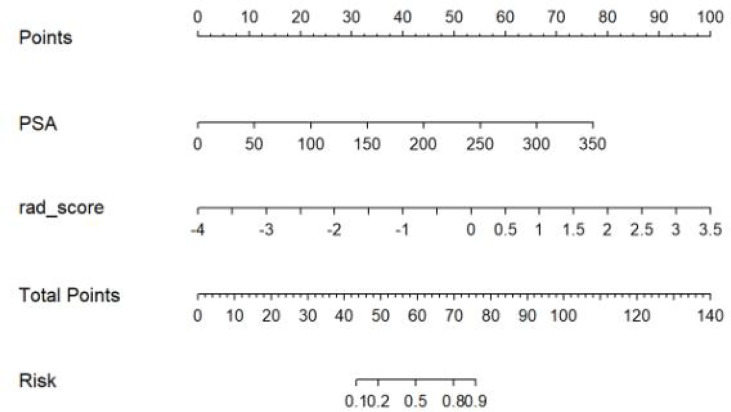
Radiomic nomogram. A nomogram for identifying prostate cancer and prostatic hyperplasia.

**Figure 4:**
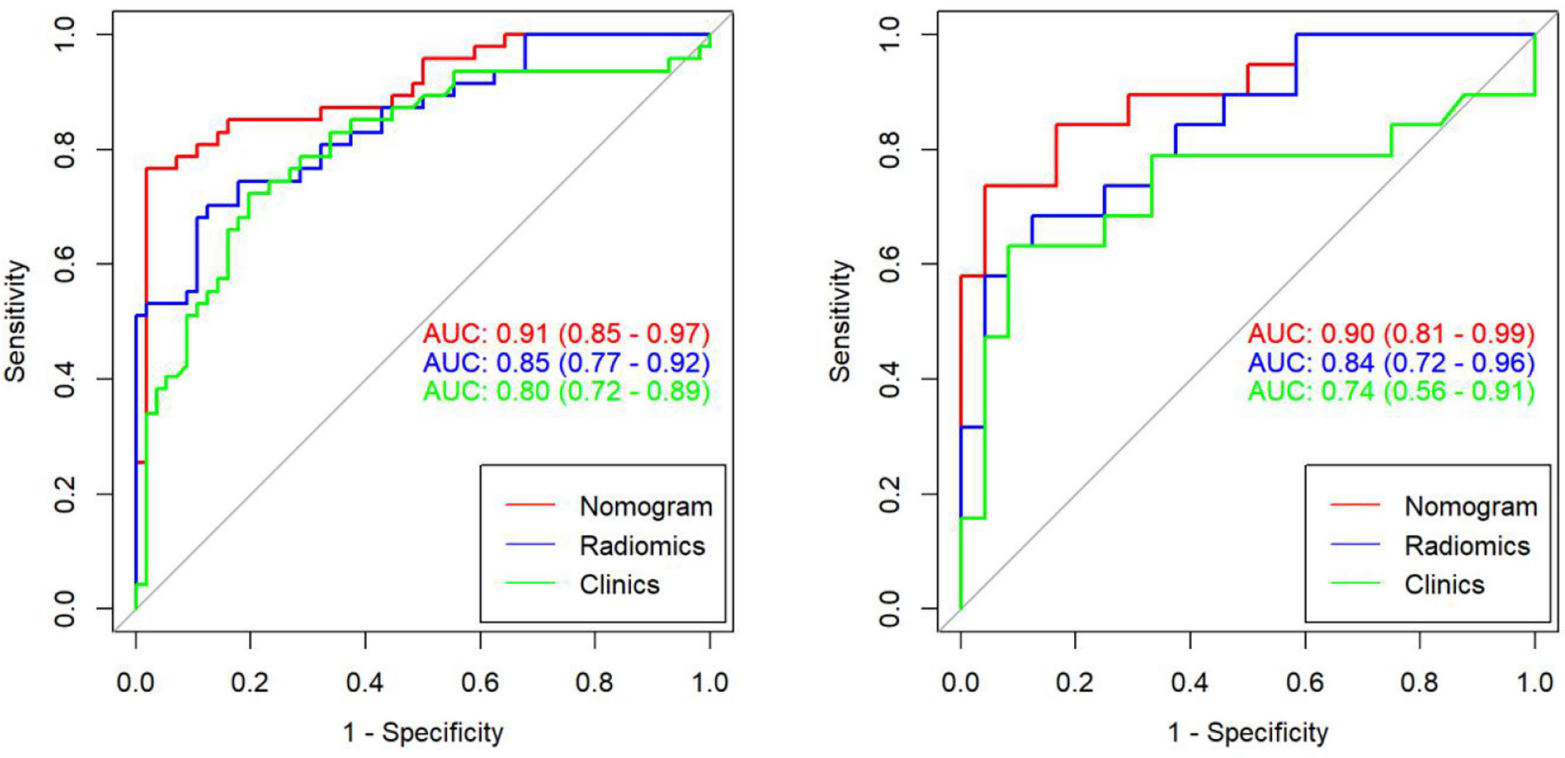
The AUC values for radiomic signatures are used in identifying prostate cancer and prostatic hyperplasia. (Left: training set; right: test set.).

We found that PSA and radiological signature could independently predict and diagnose PCa and BPH through univariate logistic regression. As shown in **Figure 3**, we used these predictors to conduct multivariate logistic regression and constructed a more robust prediction model and radiomic nomogram. The calibration curves showed good agreement between the predicted and actual pathology in the radiomic nomogram of both groups of patients. The AUC values of the nomogram-based tumor prediction in the two sets were 0.91 and 0.90, respectively. The accuracy, specificity, and sensitivity were 88.3%, 98.2%, and 76.5% for the training set and 86.0%, 82.1%, and 93.3% for the testing set, respectively (**Table 2**; **Figure 4**).

**Figure 5:**
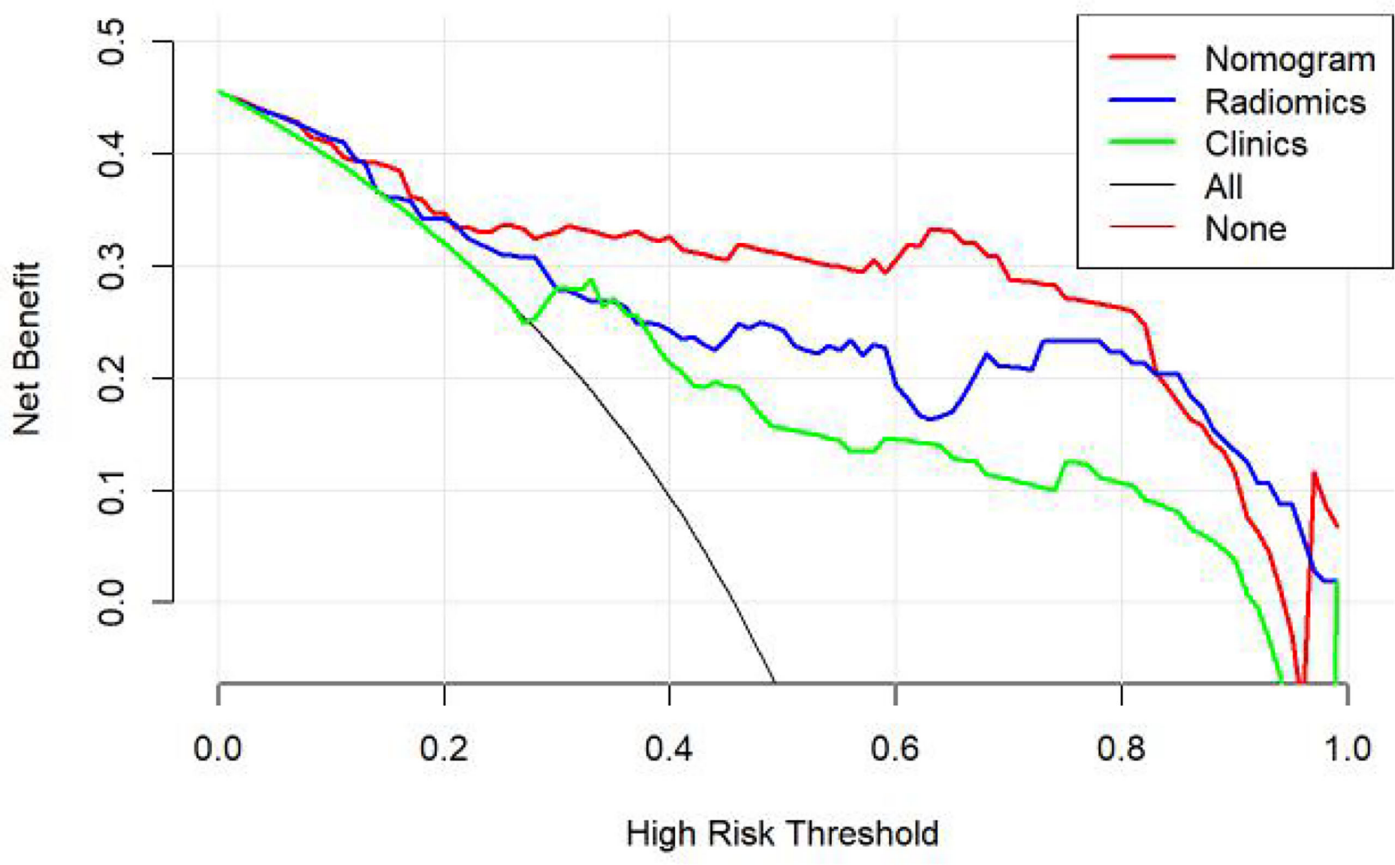
Clinical decision curve of the three models. The green, blue, and red lines correspond to the nomograms from the clinical, radiomic, and nomogram models, respectively.

According to the DeLong test, the AUCs of the models based on clinical information were significantly different from the nomogram-based ones for the training and testing sets (**Table 3**). Hence, the nomogram method was found to have a good performance on both sets. In addition, the Hosmer–Lemeshow test demonstrated no statistically significant differences among the training and testing subsets (*p* > 0.05). **Figure 5** depicts the DCA plot of the radiomic nomogram. Clearly, the plot shows that the radiomic nomogram method outperforms the clinical model for the “treat none” vs. “treat all” strategies with a treatment probability threshold ranging from 0 to 0.9.

**Table 3:** Comparison of the prediction with the radiomic nomogram, radiomic algorithm, and the clinical model.

Group	Model 1	Model 2	*p*-value
Training	Clinical	Radiomic	0.487
	Radiomics	Nomogram	0.018
	Nomogram	Clinical	0.043
Testing	Clinical	Radiomic	0.335
	Radiomics	Nomogram	0.081
	Nomogram	Clinical	0.036

## Discussion

The PI-RADS V2 is a standardized risk assessment tool for predicting the possibility of PCa. Each lesion detected was scored using three standardized MRI techniques: T2WI, DWI, and DCE-MRI. They then combine to give an overall rating category score. For some lesions that are difficult to diagnose, PI-RADS V2 can be used to provide better clinical advice. At present, the PI-RADS V2 score does not give the specific threshold of PCa diagnosis, and the clinical diagnosis threshold is usually 3 or 4. It has been suggested that A PI-RADS 3 lesion remains an equivocal lesion (16). Evaluation of clinical predictive factors in terms of clinically significant prostate cancer risk is the main aspect of helping clinicians in the biopsy decision process. One of the main limitations of PI-RADS is the high inter-reader variability impacting cancer detection (17), which led us to start our research into radiomics.

In our study, we used LASSO to finally select six types of texture feature parameters, which include simple morphological and more-comprehensive higher-order features. These higher-order features can more effectively reflect the spatial heterogeneity of tumors (18). GLCM features include two energy features and one entropy feature, in which entropy reflects tumor heterogeneity (19). The run-length matrix mainly reflects the roughness and directionality of texture. The smaller long-stroke dominating value implies the rougher texture of the images (20). Previous studies have shown that local tumor parenchyma cannot completely represent the whole tumor, and the analysis of the whole tumor can more accurately and reliably reflect the heterogeneity of the tumor (21). Therefore, in this study, we summarized ROI in all layers’ parenchyma to extract more accurate characteristic parameters. Studies have pointed out that the accuracy of MRI in different populations is unaffected (22).

Most of the previous studies used multiple sequences such as T2WI, T1, DWI, and others in radiological diagnosis to distinguish malignant and normal areas in radiomics. The results of one of the literatures (machine learning in prostate cancer identification) showed that the AUC of the combined model (T2WI, DWI) was 0.88 (23). The values for a model using IMPROD Multiparametric MRI (T2WI, DWI) findings had an AUC of 0.88 (0.84–0.92) (7). However, in clinical practice, we find that DWI images are often strongly affected by device performance, and artifacts occur frequently. Therefore, DWI images are not reliable in the delineation of lesions. At present, DWI is not a sequence routinely used in China, and its routine needs to be explored in future studies. In our study, the T2 sequence which is the most generalized and has the most stable image quality was adopted as the research sequence, and all T2 images are from the same device. However, studies on different devices and sequences need to be carried out in further research.

In recent years, radiomics have been successfully applied to oncology and extended to PCa. Previous studies have shown that there are significant differences in imaging characteristics between noncancerous and cancerous tissues, between transitional and surrounding tumors of the prostate (24, 25). Li (26) also found that compared with the evaluation of clinical risk factors, the radiomic model can improve the predictive accuracy. This indicated that the radiomic method can better reflect the risk of prostate malignant nodules than the traditional clinical features.

In our study, the nomogram was constructed using Radscore and T2 with radiological methods. Radscore is described as the probability of principal component analysis calculated from the radiomic signature, which is constructed based on nine selective radiomic features. The AUC of the radiomic signature for predicting PCa was 0.85 (training group) and 0.84 (testing group). This is different from the results provided by Zhang (27). They studied a radiomic signature based on mp-MRI to identify csPCa with an AUC of 0.95 in the training group and 0.86 in the internal validation group. This discrepancy may be due to differences in sample selection. The nomogram constructed from radiological and clinical features demonstrates good differentiation between benign and malignant prostate nodules. The AUC values of the training and testing groups are 0.91 (95% CI: 0.85–0.97) and 0.90 (95% CI: 0.81~0.99), respectively. It had a higher pathological coincidence rate. The results showed that the nomogram was effective in predicting PCa in both the training and validation groups. The ability of the nomogram to distinguish between PCa and BPH exceeds that of the radiological and clinical models. The decision curve indicates that if the patient’s threshold probability is 5% to 95%, patients can benefit more from using the radiomic nomogram in this study to predict the identification of benign and malignant nodules, and the combined model has better predictive performance than clinical risk factors or radiologic features alone. In recent years, the nomogram prediction model has been widely used in clinical medicine (27). The risk score is used to represent the risk factors of various diseases and predict the prognosis of patients. The expression of this model is clear, concise, easy to understand, and conducive to doctor–patient communication.

The PSA screening can detect well-differentiated prostate cancer but is seldom found in poorly differentiated carcinoma. In fact, the PSA can remain normal in some of the most lethal prostate cancer (28). PSA is significantly related to the differentiation of benign and malignant lesions in this study. In addition, the PSA value was the risk factor found in all clinical risk factors. This result is similar to a recent report on radiomic machine learning (29). The performance of both the radiomic signature and PSA value was high and comparable in the testing group in our study. In a previous study, Calvocoressi (29) found that the older the patient, the higher risk of histological malignancy of prostate nodules. The OR and 95% CI values of prostate tissue with a worse prognosis were 2.21 (1.30~3.76) and 1.58 (0.90–2.76), respectively, for men aged over 80 and under 70 years old. However, it is worth noting that age was not a significant factor regarding the differentiation of PCa and BPH in our study, which eliminated this variable for model development. We consider the possible cause of selection bias.

This study has several limitations: (1) Due to the small sample size, the effectiveness of the model lacks multicenter validation. (2) Manual ROI segmentation was performed on prostate tumors. This manual operation had inherent differences between and within observers. (3) Because our study was a retrospective analysis, and we excluded patients with too small lesions when delineating lesions, this may lead to patient selection bias. However, we think our results are still relatively reliable. (4) This study only established a T2W-based radiomic model, without using other sequence images, so it is impossible to know the advantages and disadvantages of each model. In the future, we will further expand the sample set and use state-of-the-art technologies such as fully automated image segmentation, deep learning, and multiparameter modeling to explore more precise diagnostic radiology. (5) Our research is single centered, which is our limitation; in the future, we want to cooperate with some international hospitals in China to do some relevant research in other populations.

## Data Availability Statement

The original contributions presented in the study are included in the article/supplementary materials. Further inquiries can be directed to the corresponding authors.

## Ethics Statement

The studies involving human participants were reviewed and approved by the ethics committee of the Jiangxi Provincial People’s Hospital. The patients/participants provided their written informed consent to participate in this study.

## Author Contributions

The authors made the following contributions: ML made the conception for this research. Data collection and analysis were performed by CW and BF. ML and SG analyzed the data and drafted the article. SN and SG reviewed/edited the manuscript. All the authors critically revised the article for important intellectual content. The authors read and approved the final manuscript.

## Funding

This work was supported by the Science and Technology Plan of Jiangxi Provincial Health Commission (grant numbers 202130023).

## Conflict of Interest

The authors declare that the research was conducted in the absence of any commercial or financial relationships that could be construed as a potential conflict of interest.

## Publisher’s Note

All claims expressed in this article are solely those of the authors and do not necessarily represent those of their affiliated organizations, or those of the publisher, the editors and the reviewers. Any product that may be evaluated in this article, or claim that may be made by its manufacturer, is not guaranteed or endorsed by the publisher.
